# Accelerated Aging in Cancer Survivors: Cellular Senescence, Frailty, and Possible Opportunities for Interventions

**DOI:** 10.3390/ijms25063319

**Published:** 2024-03-14

**Authors:** Shuo Wang, Najla El Jurdi, Bharat Thyagarajan, Anna Prizment, Anne H. Blaes

**Affiliations:** 1 Department of Laboratory Medicine and Pathology, University of Minnesota, Minneapolis, MN 55455, USA; 2 Division of Hematology, Oncology and Transplantation, University of Minnesota, Minneapolis, MN 55455, USA

**Keywords:** accelerated aging, cancer survivors, cellular senescence, frailty, anti-aging interventions

## Abstract

The population of cancer survivors has markedly increased due to the rapid improvements in cancer treatment. However, cancer survivors experience accelerated aging, which leads to chronic diseases and other age-related conditions, such as frailty. Those conditions may persist years after cancer diagnosis and treatment. Cellular senescence, a hallmark of aging, is one of the mechanisms that contribute to accelerated aging in cancer survivors. Several aging measures, including measures based on clinical markers and biomarkers, have been proposed to estimate the aging process, and some of them have shown associations with mortality and frailty in cancer survivors. Several anti-aging interventions, including lifestyle changes and anti-aging drugs, have been proposed. Future research, particularly in large-scale studies, is needed to determine the efficiency of these aging measures and anti-aging interventions before considering their application in clinics. This review focuses on the mechanisms of cellular senescence and accelerated aging in cancer survivors, assessment of the aging process using clinical markers and biomarkers, and the high prevalence of frailty in that population, as well as possible opportunities for anti-aging interventions. A deeper understanding of aging measures and anti-aging interventions in cancer survivors will contribute to the development of effective strategies to mitigate accelerated aging in cancer survivors and improve their quality of life.

## 1. Introduction

There is a growing concern about accelerated aging among the rapidly increasing number of cancer survivors. Cancer survivors experience premature mortality, frailty, sarcopenia, cognitive impairment, and other age-related diseases and disabilities earlier in life than similarly aged individuals who have no history of cancer [1,2,3]. These accelerated aging phenotypes lead to a lower quality of life and increased healthcare expenses for cancer survivors. Therefore, there is a need for a better understanding of the biological mechanisms involved in the aging process among cancer survivors. In addition, there is a need for biomarkers that can be used to quantify the aging process in cancer survivors and predict the risk of accelerated aging phenotypes. Aging biomarkers may serve as targets for anti-aging interventions. Moreover, these biomarkers could be employed in intervention trials or studies to assess the efficacy of anti-aging interventions in cancer survivors.

**Figure 1:**
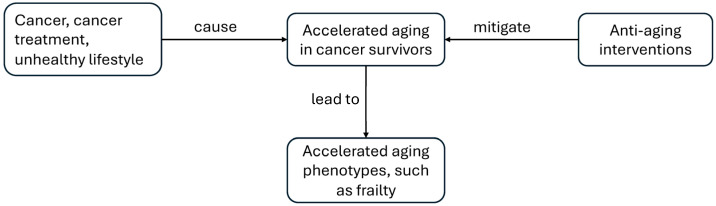
Possible mechanisms of accelerated aging and anti-aging intervention among cancer survivors.

Accelerated aging in cancer survivors can be attributed to cancer itself and cancer treatment [1,3,4,5,6]. The accumulation of stress induced by cancer and its treatment may incite hallmarks of aging, such as cellular senescence, inflammation, telomere shortening, epigenetic alterations, and mitochondrial dysfunction [2,7]. These hallmarks of aging are interconnected with each other and contribute to accelerated aging [8]. In addition, accelerated aging in cancer survivors can be caused by unhealthy lifestyles, such as cigarette smoking and a lack of physical activity [4,9,10]. This review focuses on the mechanisms of cellular senescence and accelerated aging in cancer survivors, assessment of the aging process using clinical makers and biomarkers, and the high prevalence of frailty in that population, as well as possible opportunities for anti-aging interventions (Figure 1).

## 2. Cellular Senescence and Accelerated Aging in Cancer Survivors

Cellular senescence, which is irreversible cell cycle arrest, can be induced by various stimuli, such as DNA damage, cellular stress, telomere shortening, and the activation of oncogenes [11,12]. Initially, cellular senescence was regarded as a tumor-suppressing mechanism by preventing cancer cells from proliferating [13]. Therefore, many commonly used cancer treatments, such as chemotherapeutic agents and radiation therapy, induce cellular senescence in tumor cells [11,14,15,16,17,18]. However, these treatment modalities may also induce cellular senescence in adjacent normal cells and result in the generation of senescent cells [19].

In addition to senescent cells induced by cancer treatment, as people grow older, senescent cells accumulate in the human body. These cells eventually stop multiplying but do not undergo cell death [20]. Senescent cells release senescence-associated secretory phenotype (SASP) factors, including proinflammatory cytokines, chemokines, growth factors, and other factors that can trigger inflammation [20,21,22,23]. Previous research using mouse models has suggested that senescent cells induced by chemotherapy persist and contribute to both local and system inflammation, as measured by the increased expression of SASP factors in tissue and blood [17].

As the immune system becomes less effective with age, the accumulation of senescent cells can affect an individual’s ability to resist age-related conditions [19,23]. Therefore, cellular senescence has been linked to multiple age-related conditions, such as frailty, cardiovascular disease, and cognitive impairment [24,25,26]. Previous studies have used mouse models to study senescent cells [16,27]. For example, a previous study found that, in mouse models, transplantation of a small number of senescent cells into young mice induced physical dysfunction as measured by reduced walking speed, muscle strength, physical endurance, food intake, and body weight [16].

Given the important role of cellular senescence in accelerated aging among cancer survivors, it is essential to understand the interplay between cellular senescence and the aging process. This understanding will help to develop targeted interventions that can mitigate accelerated aging in cancer survivors and improve the overall health and quality of life of that population.

## 3. Assessment of the Aging Process

How far an individual is into the aging process, or an individual’s extent of aging, is unique to that individual and cannot be easily estimated by chronological age, especially for cancer survivors. Individuals with and without a history of cancer at the same chronological age may experience very different physiological dysfunctions (i.e., have different biological ages). Biological age, according to Baker and Sprott’s definition, is characterized by the “biological parameter[s] of an organism, either alone or in some multivariate composite that will, in the absence of disease, better predict functional capability at some late age than will chronological age” [28].

To better estimate the aging process of cancer survivors, a variety of clinical assessments, such as the geriatric assessment, the Karnofsky Performance Scale (KPS), and the Eastern Cooperative Oncology (ECOG) scale, have been implemented in clinics [1,3,29]. These clinical assessments can predict cancer treatment toxicity and survival and identify cancer survivors who need interventions for improved outcomes [29,30]. One recently published meta-analysis of six randomized control trials found that cancer patients who received comprehensive geriatric assessment after chemotherapy had significantly lower treatment-related toxicity (grade 3+ toxicity) compared to those patients who received standard care after treatment [31].

### 3.1. Clinical Marker-Based and Biomarker-Based Aging Measures

In addition to clinical assessments, several clinical marker-based and biomarker-based aging measures, including clinical marker-based multidomain aging constructs, aging clocks, *p16^INK4a^* expression, senescence-associated secretory phenotype (SASP) proteins, and inflammatory markers, have been proposed to quantify an individual’s biological age. Compared to clinical assessments, these measures offer the advantage of being easily and less invasively collected. In the following section, we present several clinical marker-based and biomarker-based aging measures that have been tested in observational studies (Table 1).

### 3.2. Clinical Marker-Based Aging Measures

Levine et al. developed two multidomain aging constructs comprising biochemical, hematological, and physical markers associated with the aging process and mortality [32,33]. The first multidomain aging construct is biological age (BioAge), which was developed using the Klemara Doubal algorithm. BioAge is constructed from ten clinical markers and physiological measures known to be associated with the aging process, including systolic blood pressure (SBP), total cholesterol, fasting glucose, cytomegalovirus (CMV) infection, C-reactive protein (CRP), serum creatinine, blood urea nitrogen (BUN), alkaline phosphatase, albumin, and peak flow measurement [32]. The other construct is Phenotypic Age (PhenoAge). PhenoAge is constructed using chronological age as well as nine clinical biomarkers associated with mortality, including albumin, creatinine, glucose, log-transformed CRP, lymphocyte percent, mean cell volume, red blood cell distribution width, alkaline phosphatase, and white blood cell count [33]. Both BioAge and PhenoAge have been found to be significantly associated with an increased risk of mortality in previous studies [34,35], indicating their capacity to estimate an individual’s biological age. For example, in our previous study in the Health and Retirement Study (HRS), we found that PhenoAge was associated with an increased risk of all-cause mortality over four years of follow-up in both cancer survivors [hazard ratio (HR) per one standard deviation (SD) = 1.47, 95% confidence interval (CI): 1.14–1.90] and individuals without cancer [HR per one SD = 1.37, 95% CI: 1.19–1.58] [35]. BioAge was associated with the risk of all-cause mortality in individuals without cancer [HR per 1 SD = 1.32, 95% CI: 1.10–1.59], while the association between BioAge and all-cause mortality in cancer survivors was positive but did not reach statistical significance [HR per one SD = 1.14, 95% CI: 0.95–1.37] [35].

### 3.3. Aging Clocks

Within the last decade, researchers have developed aging measures called aging clocks using DNA methylation (called “epigenetic clocks”) [33,36,37,38,39], proteomics (called “proteomic aging clocks”) [40,41,42,43], and other biomarkers. Most aging clocks were trained against chronological age or mortality. Aging clocks can be used to identify individuals whose biological age is higher than their chronological age (the positive deviation is called age acceleration) [44] and predict their future risk of mortality and age-related diseases.

The most commonly used aging clocks in published studies are epigenetic clocks. Several epigenetic clocks, including the Horvath clock [36], Hannum clock [37], DNAm PhenoAge [33], and GrimAge [38], have been calculated in observational studies and their associations with the risk of mortality have been examined [35,38]. In addition to the above epigenetic clocks, a new aging construct based on DNA methylation—DunedinPACE—has been developed [39]. This aging construct measures the pace of aging, i.e., “years of physiological decline occurring per 12 months of calendar time” [39], rather than an individual’s biological age in years. In our recent HRS study, we found that cancer survivors tended to have a biological age, estimated using the Horvath clock, Hannum clock, DNAm PhenoAge, and GrimAge, higher than their chronological age. In addition, compared to individuals without cancer, cancer survivors were more likely to have a faster pace of aging [35]. We also found that age acceleration for the Hannum clock and GrimAge was significantly associated with an increased risk of all-cause mortality in both cancer survivors and individuals without cancer, with the strongest association observed for GrimAge and the risk of all-cause mortality in cancer survivors [HR per one SD = 2.03, 95% CI: 1.28–2.06] [35]. Age acceleration for the Horvath clock and DNAm PhenoAge was significantly associated with the risk of all-cause mortality in cancer survivors only, while the pace of age was significantly associated with the risk of all-cause mortality in individuals without cancer only [35]. Although DNA methylation-based aging constructs have been commonly used in observational studies and hold promise for mortality prediction, there is a lack of understanding of the underlying mechanisms of aging-related changes in DNA methylation sites. The aspects of aging reflected by these constructs remain unclear [45].

With assays measuring thousands of proteins simultaneously, such as the SomaScan assay [46] and the Olink assay [47], now available, it is possible to construct proteomic aging clocks (PACs) using circulating proteins. A key strength of PACs is that proteins, serving as an intermediate phenotype, are the most proximal to age-related diseases. Consequently, proteins comprising PACs may provide more accurate information on aging and age-related pathologies [40,48]. In addition, proteins are the targets in 96% of FDA-approved drugs [49]. Therefore, proteins comprising PACs may hold promise as targets of anti-aging drugs. Targeting age-related processes or pathologies instead of a single disease is advantageous, as this approach may reduce the development or progression of multiple age-related diseases at the same time [50]. In our recent study, we constructed midlife and late-life PACs in the Atherosclerosis Risk in Communities (ARIC) study (called the midlife ARIC PAC and the late-life ARIC PAC) [43] and also computed the PACs developed by Tanaka (2018) [40], Lehallier (2020) [41], and Sathyan (2020) [42]. We found that all these PACs were significantly associated with an increased risk of mortality in the general population [43]. For example, one SD increase in age acceleration for the midlife ARIC PAC was associated with a 38% increased risk of all-cause mortality in midlife participants [95% CI: 1.34–1.42]. One SD increase in age acceleration for the late-life ARIC PAC was associated with a 65% increased risk of all-cause mortality in late-life participants [95% CI: 1.52–1.79] [43]. Table 2 presents nine proteins and their mechanisms as documented in STRING (https://string-db.org/, accessed on 6 March 2024). These are common proteins included in published PACs that were developed using the SomaScan assays.

### 3.4. p16^INK4a^ Expression

p16^INK4a^ is a cell cycle protein that slows cell division by limiting the progression from the G1 phase to the S phase of the cell cycle and has been linked to aging and senescence [51]. The expression of *p16^INK4a^* in peripheral blood T lymphocytes increases exponentially with chronological age [52], making it a potential biological age estimator. Several studies have compared *p16^INK4a^* expression in cancer survivors to the expression in age-matched individuals without cancer. For instance, Smitherman et al. found that the mean of the *p16^INK4a^* expression in 60 childhood, adolescent, and young adult cancer survivors (aged 18–29 years) was higher than the mean expression in 29 age-matched individuals without cancer (*p*-value < 0.01) [53]. Similarly, a study of testicular cancer survivors treated with chemotherapy found higher *p16^INK4a^* expression in 16 cancer survivors (aged 24–54 years) compared to the expression in 16 age-matched controls without a history of cancer (*p*-value = 0.048) [54]. Previous studies have also investigated the change in *p16^INK4a^* expression in cancer survivors after receiving treatment. For example, in a study involving 33 females with stage I and II breast cancer, Sanoff et al. found that the expression of *p16^INK4a^* increased immediately after receiving chemotherapy and remained elevated even 12 months after receiving treatment [55].

### 3.5. Senescence-Associated Secretory Phenotype (SASP) Proteins

SASP proteins released by senescent cells may have the potential to be used as biomarkers for biological age, given that cellular senescence is one of the mechanisms that contribute to aging [25,40]. Several studies proposed different lists of circulating SASP proteins. For example, Basisty et al. reported 177 SASP Atlas proteins, which is the current largest list of SASP proteins [56]. Tanaka et al. described a list of 72 SASP proteins based on previous literature. Among these 72 SASP proteins, growth differentiation factor 15 (GDF15), tumor necrosis factor (TNF) receptor superfamily member 6 (FAS), growth-regulated alpha protein (CXCL1), epidermal growth factor receptor (EGFR), insulin-like growth factor-binding protein 3 (IGFBP3), macrophage metalloelastase (MMP12), and vascular endothelial growth factor A (VEGFA) were included in the PAC developed by Tanaka (2018) [40]. Schafer et al. identified a panel of seven SASP proteins, including GDF15, FAS, osteopontin (OPN), TNF receptor 1 (TNFR1), ACTIVIN A, chemokine (C-C motif) ligand 3 (CCL3), and interleukin-15 (IL-15). This panel was found to predict adverse events markedly better than a single SASP protein or chronological age [25]. In a recent meta-analysis of the Baltimore Longitudinal Study of Aging (BLSA)/GESTALT and the Invecchiare in Chianti (Aging in the Chianti Area, InCHIANTI) study, Evans et al. [57] examined 77 SASP proteins, which were proteins measured in the SomaScan assay (V.2) out of the 177 SASP Atlas proteins [56]. Among these proteins, GDF-15, insulin-like growth factor-binding protein 2 (IGFBP2), and Cystatin-C showed the most significant associations with age and demonstrated associations with poor physical function, characterized by lower grip strength or slower gait speed [57].

In addition to the above aging measures, chronic inflammatory markers such as CRP and interleukin-6 (IL-6) have been extensively studied as chronic inflammation is a hallmark of aging [8]. Increased levels of CRP and IL-6 have been shown to be associated with the risk of mortality and frailty [58,59]. Furthermore, metabolites have the potential to be used as aging markers [60]. 

In summary, multiple clinical marker-based and biomarker-based aging measures have been developed. Some of these aging measures have been tested in cancer survivors and demonstrated promise in predicting biological age. Different aging measures may capture different aspects of aging and result in variations in predicting biological age. Further research is necessary to understand how these aging measures can be effectively applied clinically and in clinical trials to estimate the aging process in cancer survivors.

### 3.6. Application of Assessment of Biological Age in Cancer Survivors

Currently, chronological age is a common parameter used in cancer treatment guidelines. However, chronological age does not account for the considerable variation in the ability of cancer survivors of the same chronological age to tolerate treatment toxicity. Assessing biological age before treatment may better predict the risk of treatment toxicity and guide physicians in tailoring cancer treatments for improved outcomes. In addition, assessing biological age after treatment in cancer survivors may predict their future risk of mortality and age-related diseases, such as frailty, and inform physicians and caregivers about the necessity of anti-aging care. In summary, by incorporating biological age into the evaluation process of cancer treatment and care plans, healthcare professionals can gain a more comprehensive understanding of cancer survivors’ overall health. This, in turn, enables them to make informed decisions regarding personalized treatment plans and supportive care based on the needs of each cancer survivor.

## 4. Frailty in Cancer Survivors

Frailty, a common geriatric syndrome, affects approximately 10% of community-dwelling elderly people aged 65 years and older [61]. Frailty is characterized by age-related declines in physiologic reserve and function, which lead to increased vulnerability to adverse outcomes [62]. Notably, the prevalence of frailty in cancer survivors is much higher than the prevalence in the general population. As reported in the review by NESS and Wogksch (2020), the prevalence of frailty ranged from 7.9% to 47% in survivors of childhood cancer in their third and fourth decades of life and from 9.1% to 59% in adult cancer survivors [63].

Frailty is not solely defined based on physical capabilities but is a multifaceted condition that includes several components, such as physical health and psychological and social factors [64]. A variety of tools have been employed to assess frailty (Table 3). The Fried frailty phenotype, a commonly used tool to assess frailty, was initially described in the Cardiovascular Health Study (CHS). The Fried frailty phenotype consists of five criteria: unintentional weight loss, self-reported exhaustion, weakness, slow walking speed, and low physical activity [65]. Individuals are categorized as frail if three or more criteria are present, pre-frail if one or two criteria are present, and robust if no criteria are present [65]. In addition to the frailty phenotype, frailty has been assessed using an accumulation of deficits approach. A frailty index is based on the accumulation of deficits across various physiological and mental domains and is calculated as the sum of deficits accrued by an individual divided by the total number of deficits composing the index [66]. Recently, an electronic frailty index based on demographics, vital signs, smoking status, diagnosis, select outpatient laboratory measurements, and functional information has been proposed [67,68]. Other measures, including grip strength, the timed up-and-go, and the 6 min walk test, have also been used in studies to assess frailty [69,70,71].

Managing frailty in cancer survivors poses a great challenge for healthcare providers, as it is associated with premature mortality, functional decline, poor quality of life, and other adverse health outcomes [63,72]. Given the high prevalence of frailty and the challenges in its management among cancer survivors, there is a need to identify biomarkers that can predict frailty in that population and enable timely interventions. Several studies have examined the associations between biomarker-based aging measures and frailty in cancer survivors (Table 1). For example, in our previous study, which included 55 bone marrow transplant survivors and 43 breast cancer survivors who had received chemotherapy, we found that frail and pre-frail survivors, defined based on the Fried frailty phenotype, were more likely to have a higher *p16^INK4a^* expression compared to robust survivors (*p*-value < 0.01) [72]. Similarly, a study of 60 childhood, adolescent, and young adult cancer survivors by Smitherman et al. found that *p16^INK4a^* expression was, on average, higher among frail cancer survivors compared to the expression in pre-frail (*p*-value = 0.23) and robust (*p*-value = 0.055) survivors [53]. A recent systematic review indicated that multiple studies have found a significant association between higher levels of IL-6 and frailty in patients with solid tumors [73]. These results suggest the potential of IL-6 as a biomarker for frailty in cancer survivors. That review also identified the neutrophil–lymphocyte ratio (NLR) and the Glasgow Prognostic Score (GPS), which is the ratio between CRP and albumin, as potential biomarkers for frailty in patients with solid tumors [73]. While these findings suggest the potential use of biomarker-based aging measures for frailty prediction in cancer survivors, the majority of these studies have relatively small sample sizes (less than 100 participants). Larger studies investigating the association between aging measures and frailty in cancer survivors are needed to support the conclusions.

## 5. Possible Opportunities for Anti-Aging Interventions

The Geroscience hypothesis states that an individual’s extent of aging can be modified by strategies targeting the hallmarks of aging. This hypothesis offers an exciting opportunity for the development of anti-aging interventions. By implementing anti-aging interventions across cancer diagnosis, active cancer treatment, and post-treatment phases, we may not only decelerate cancer survivors’ aging process but may also anticipate a delay or prevention of the onset of multiple age-related conditions [50,74]. A growing body of literature suggests that an individual’s aging process can be slowed through changes in lifestyle and the use of anti-aging drugs [50,75] (Table 4).

### 5.1. Lifestyle Changes

#### 5.1.1. Physical Exercise

Physical exercise may serve as a cost-effective and generally low-risk preventive strategy to mitigate accelerated aging [78,79]. There is evidence suggesting that regular exercise can attenuate multiple hallmarks of aging, including cellular senescence [80]. In 2019, the report published by the American College of Sports Medicine Roundtable provided evidence that exercise improved cancer-related health outcomes [76]. Given the multifaced benefits of exercise, there is an opportunity to reduce accelerated aging in cancer survivors by incorporating physical exercise.

#### 5.1.2. Diet and Nutrition

During active cancer treatment, some patients may experience weight loss due to side effects of cancer treatment, such as reduced appetite, vomiting, mouth sores, and changes in the way food tastes or smells. This can directly impact their nutritional status and potentially lead to malnutrition, characterized by inadequate nutrient intake [81,82]. In cancer survivors, malnutrition may reduce their tolerance to cancer treatment and elevate the risk of adverse outcomes, such as unwanted treatment side effects, lower quality of life, and a shorter survival time [83,84]. Conversely, the high prevalence of obesity in the United States contributes to an increased incidence of obesity among cancer survivors. Obesity, a well-known risk factor for various age-related diseases, can obscure signs of malnutrition [85,86]. Therefore, screening for malnutrition in cancer patients is crucial to identify those at a higher risk, even within the context of obesity [86]. Providing nutrition counseling and care for cancer survivors may facilitate more successful treatment outcomes, improve their quality of life, and decrease the risk of mortality [86]. Nutritional support may be particularly important for elderly cancer survivors, as their nutritional needs may differ substantially from those of younger cancer survivors [81].

### 5.2. Anti-Aging Drugs: Senolytics and Senomorphics

Cellular senescence has been recognized as one of the mechanisms that contribute to accelerated aging in cancer survivors. It has been proposed that clearing senescent cells after cancer treatment may potentially mitigate accelerated aging and prevent or delay the onset of age-related conditions in cancer survivors [18,77]. Senolytics are a class of drugs designed to selectively kill senescent cells or induce the death of senescent cells. A recent paper published by Zhang et al. reported a list of current senolytics, including cardiac glycosides, galactose-modified senolytic pro-drugs, and dasatinib and quercetin [77]. Senolytics have been shown to decelerate the aging process in animal models. For example, in mouse models, the clearance of senescent cells by senolytics has been found to reverse age-related conditions, such as frailty [16]. However, even after the elimination of senescent cells, the senescence-associated secretory phenotype (SASP) factors released by these cells may persist. Another class of drugs, known as senomorphics, has been shown to suppress the detrimental effect of SASP factors secreted by senescent cells without clearing senescent cells [5,77]. Senomorphics include rapamycin, metformin, resveratrol, and aspirin [77]. In a recently published study of chemotherapy-induced senescent endothelial cells, the group treated with metformin had lower levels of SASP factors compared to the group without metformin treatment [87]. Multiple clinical trials that aim to evaluate the impact of the clearance of senescence in cancer survivors are currently in the recruiting phase. These trials include investigations in adult survivors of childhood cancer (NCT04733534), hematopoietic stem cell transplant survivors (NCT02652052), and older breast cancer survivors (NCT05595499) [4].

Although several anti-aging interventions for cancer survivors have been proposed, there are challenges that need to be addressed. Long-term safety is one of the concerns associated with senolytics and senomorphics. For example, long-term use of aspirin increases the risk of gastrointestinal bleeding [88], and some senolytics may cause pulmonary hypertension [89]. Furthermore, although several biomarkers of aging have been proposed, we currently do not know which is the best measure to monitor the effectiveness of these drugs. Additional research is needed to determine the safety and efficacy of anti-aging interventions and comprehensively understand how anti-aging interventions can be integrated with cancer therapy to reduce accelerated aging in cancer survivors.

## 6. Future Directions

The challenge of accelerated aging in cancer survivors has become a significant concern within the rapidly increasing population of cancer survivors in the United States. As mentioned earlier, we currently do not know which is the best aging measure, despite several aging measures having been proposed. Future studies are encouraged to investigate the effectiveness of these published aging measures and identify the optimal one or explore alternative measures. In addition, the majority of existing results on aging among cancer survivors are derived from studies of childhood cancer survivors. Future studies are encouraged to investigate the aging process among survivors of adult cancer. Furthermore, besides cancer itself and cancer treatment, genetic factors may contribute to aging [90,91]. However, there is a limited number of studies that have discussed the association between genetic factors and aging among cancer survivors. Understanding the effect of genes on the aging process in cancer survivors may inform genetic testing and treatment decisions [92]. For example, cancer patients with TP53 mutations are recommended to avoid radiation therapy, which increases the risk of developing secondary cancer [93]. Future studies are encouraged to explore the association between genetic factors and aging among cancer survivors.

## Figures and Tables

**Table 1:** Summary of clinical marker-based and biomarker-based aging measures.

Aging Measures	Description of Aging Measures and Their Associations with Mortality and Frailty in Cancer Survivors
Clinical marker-based aging measures	
Biological age (BioAge), Phenotypic Age (PhenoAge)	They comprise biochemical, hematological, and physical markers associated with the aging process and mortality and have been found to be associated with the risk of mortality in cancer survivors.
Aging clocks	
Epigenetic clocks	They are constructed using DNA methylation and have been found to be associated with the risk of mortality in cancer survivors. However, there is a lack of understanding of the underlying mechanisms of aging-related changes in DNA methylation sites.
Proteomic aging clocks (PACs)	They are constructed using circulating proteins. Proteins, as an intermediate phenotype, are the most proximal to age-related diseases. Therefore, proteins comprising PACs may provide more accurate information on aging and age-related pathologies.
Single marker-based aging measures	
*p16^Ink4a^* expression	*p16^INK4a^* expression in peripheral blood T lymphocytes increases exponentially with chronological age. *P16^INK4a^* expression has been found to be associated with frailty in cancer survivors.
IL-6	IL-6 is an inflammation maker and has been found to be associated with frailty in cancer survivors.
Senescence-associated secretory phenotype (SASP) proteins	
Several lists of SASP proteins have been proposed	SASP proteins released by senescent cells may have the potential to be used as biomarkers for biological age, given that cellular senescence is one of the mechanisms that contribute to aging. To our knowledge, no studies have examined whether SASP proteins can predict the risk of mortality and frailty in cancer survivors.

**Table 2:** Proteins commonly included in proteomic aging clocks (PACs).

Protein Name	Uniprot ID	Mechanisms ^a^ of Proteins	Included in Which PACs
Pleiotrophin (PTN)	P21246	Regulates many processes like cell proliferation, cell survival, cell growth, cell differentiation, and cell migration in several tissues, namely neurons and bone.	Lehallier’s, Tanaka’s, and Sathyan’s PACs, midlife ARIC PAC, and late-life ARIC PAC
A disintegrin and metalloproteinase with thrombospondin motifs 5 (ADAMTS5)	Q9UNA0	Plays an important role in connective tissue organization, development, inflammation, arthritis, and cell migration.	Lehallier’s, Tanaka’s, and Sathyan’s PACs, midlife ARIC PAC, and late-life ARIC PAC
Macrophage metalloelastase (MMP12)	P39900	May be involved in tissue injury and remodeling.	Lehallier’s, Tanaka’s, and Sathyan’s PACs, midlife ARIC PAC, and late-life ARIC PAC
Cell adhesion molecule-related/down-regulated by oncogenes (CDON)	Q4KMG0	Promotes differentiation of myogenic cells (by similarity).	Lehallier’s, Tanaka’s, and Sathyan’s PACs, midlife ARIC PAC, and late-life ARIC PAC
Growth/differentiation factor 15 (GDF15)	Q99988	Regulates food intake, energy expenditure, and body weight in response to metabolic and toxin-induced stresses.	Lehallier’s, Tanaka’s, and Sathyan’s PACs and midlife ARIC PAC
Immunoglobulin superfamily containing leucine-rich repeat protein 2 (ISLR2)	Q6UXK2	Required for axon extension during neural development.	Lehallier’s, Tanaka’s, and Sathyan’s PACs and midlife ARIC PAC
Kallikrein-7 (KLK7)	P49862	Could play a role in the activation of precursors to inflammatory cytokines.	Lehallier’s, Tanaka’s, and Sathyan’s PACs and midlife ARIC PAC
Lactoperoxidase (LPO)	P22079	May contribute to airway host defense against infection.	Lehallier’s, Tanaka’s, and Sathyan’s PACs and midlife ARIC PAC
R-spondin-4 (RSPO4)	Q2I0M5	Activator of the canonical Wnt signaling pathway by acting as a ligand for LGR4-6 receptors. Also regulates the canonical Wnt/beta-catenin-dependent pathway and non-canonical Wnt signaling by acting as an inhibitor of ZNRF3.	Lehallier’s, Tanaka’s, and Sathyan’s PACs and midlife ARIC PAC

^a^ Mechanisms of proteins documented in STRING (https://string-db.org/, accessed on 6 March 2024).

**Table 3:** Frailty measures used in previous studies.

Measures commonly used in published studies	
Measure	Description
The Fried frailty phenotype	Consists of five criteria: unintentional weight loss, self-reported exhaustion, weakness, slow walking speed, and low physical activity [65]. Individuals are categorized as frail if three or more criteria are present, pre-frail if one or two criteria are present, and robust if no criteria are present [65].
Frailty index	A frailty index is based on the accumulation of deficits across various physiological and mental domains and is calculated as the sum of deficits accrued by an individual divided by the total number of deficits composing the index [66].
Other measures used in previous studies	
Electronic frailty index (based on demographics, vital signs, smoking status, diagnosis, select outpatient laboratory measurements, and functional information) [67,68], grip strength, the timed up-and-go, and the 6 min walk test [69,70,71].	

**Table 4:** Evidence, hypotheses, and mechanisms of anti-aging interventions.

Lifestyle Changes	Evidence or Hypothesis
Physical exercise	The report published by the American College of Sports Medicine Roundtable in 2019 provided evidence that exercise improved cancer-related health outcomes [76].
Diet and nutrition	Providing nutrition counseling and care for cancer survivors may enhance treatment outcomes, improve their quality of life, and decrease the risk of adverse outcomes.
**Anti-Aging Drugs**	**Mechanism**
Senolytics	Senolytics selectively kill senescent cells or induce the death of senescent cells [77].
Senomorphics	Senomorphics suppress the detrimental effect of SASP factors secreted by senescent cells without clearing senescent cells [77].

## Data Availability

Not applicable.
